# Platelet and epithelial cell interations can be modeled in cell culture, and are not affected by dihomo-gamma-linolenic acid

**DOI:** 10.1371/journal.pone.0309125

**Published:** 2024-08-27

**Authors:** Zitha Redempta Isingizwe, Laura F. Mortan, Doris Mangiaracina Benbrook

**Affiliations:** 1 Department of Pharmaceutical Sciences, College of Pharmacy, University of Oklahoma Health Sciences Center, Oklahoma City, OK, United States of America; 2 Division of Gynecologic Oncology, Department of Obstetrics and Gynecology, Stephenson Cancer Center, University of Oklahoma Health Sciences Center, Oklahoma City, OK, United States of America; 3 Department of Pathology, Stephenson Cancer Center, University of Oklahoma Health Sciences Center, Oklahoma City, OK, United States of America; Southern Illinois University School of Medicine, UNITED STATES OF AMERICA

## Abstract

Increasing evidence is implicating roles for platelets in the development and progression of ovarian cancer, a highly lethal disease that can arise from the fallopian tubes, and has no current method of early detection or prevention. Thrombosis is a major cause of mortality of ovarian cancer patients suggesting that the cancer alters platelet behavior. The objective of this study was to develop a cell culture model of the pathological interactions of human platelets and ovarian cancer cells, using normal FT epithelial cells as a healthy control, and to test effects of the anti-platelet dihomo-gamma-linolenic acid (DGLA) in the model. Both healthy and cancer cells caused platelet aggregation, however platelets only affected spheroid formation by cancer cells and had no effect on healthy cell spheroid formation. When naturally-formed spheroids of epithelial cells were exposed to platelets in transwell inserts that did not allow direct interactions of the two cell types, platelets caused increased size of the spheroids formed by cancer cells, but not healthy cells. When cancer cell spheroids formed using magnetic nanoshuttle technology were put in direct physical contact with platelets, the platelets caused spheroid condensation. In ovarian cancer cells, DGLA promoted epithelial-to-mesenchymal (EMT) transition at doses as low as 100 μM, and inhibited metabolic viability and induced apoptosis at doses ≥150 μM. DGLA doses ≤150 μM used to avoid direct DGLA effects on cancer cells, had no effect on the pathological interactions of platelets and ovarian cancer cells in our models. These results demonstrate that the pathological interactions of platelets with ovarian cancer cells can be modeled in cell culture, and that DGLA has no effect on these interactions, suggesting that targeting platelets is a rational approach for reducing cancer aggressiveness and thrombosis risk in ovarian cancer patients, however DGLA is not an appropriate candidate for this strategy.

## Introduction

Ovarian cancer is the most lethal gynecological cancer and the 5^th^ leading cause of cancer-related deaths in women in the United States [1]. This is due to the lack of early detection, while late detection is often associated with advanced stage disease that has already spread to neighboring organs in the peritoneal cavity. Most importantly, there are no approved standard methods for the prevention of ovarian cancer.

Multiple roles of platelets in many inflammatory diseases and cancers have been reported, making them a targetable agent for cancer prevention. They are rich in platelet derived growth factor (PDGF), which provides self-sufficiency in cell growth, and transforming growth factor-β (TGF-β), a cytokine that induces epithelial-to-mesenchymal transition, hence facilitating primary tumor invasion to neighboring tissues and metastasis to distant tissues [2–4]. Platelets have been shown to help cancer cells evade anoikis by activating yes-associated protein 1 (YAP1) signaling [5]. Cyclooxygenase-2 (COX-2) is constitutively expressed in platelets, COX-2 products are well-characterized for their promotion of cancer, especially prostaglandin E2 (PGE2), a metabolite of arachidonic acid that promotes inflammation and tumor growth [6]. In situations involving an uncompromised immune system, cells with damaged DNA are removed from the system. However, when platelets are recruited at a tumor site, they have been shown to form a mesh that surrounds the cell mass and protects the cancer against infiltration of immune cells, such as natural killer cells [7]. This mesh formation also protects circulating tumor cells against blood pressure-induced stress [8]. The potential role of platelets in deregulation of cellular energetics has not yet been reported.

Activation of platelets at an actively proliferating site provides further support for tumors by releasing angiopoietin and vascular endothelial growth factor (VEGF), which helps in establishment of new blood vessels, thereby fueling the tissues with oxygen and nutrients required for growth and proliferation. Thus, platelets promote cancer by facilitating cancer cell evasion of apoptosis and the immune system, and insensitivity to anti-growth signals, while stimulating angiogenesis to provide nutrients that support the limitless replicative potential of cancer cells, thereby successfully establishing a stable tumor.

This knowledge of platelet promotion of cancer has inspired previous studies using anti-platelet agents, such as selective COX-2 inhibitors or aspirin, for cancer prevention purposes. Low-dose aspirin is an acceptable method for colorectal cancer prevention [9], but its role in ovarian cancer is yet to be justified as there are opposing reports regarding this matter. Aspirin was found to partially inhibit platelet-mediated cancer cells invasion [10] and proliferation [11], while other antiplatelet agents including a COX-2 inhibitor, an EP3 (PGE2 receptor) inhibitor and a P2Y12 receptor antagonist were found to interfere with platelet promotion of cancer [12]. A very well-characterized process of tumor cell-induced platelet aggregation (TCIPA), which is believed to contribute to higher levels of thrombotic events in cancer patients, was found to be inhibited by an aspirin nicotinate prodrug, but not aspirin itself [13]. TCIPA inhibition was associated with reduced metastasis and cancer cell proliferation. Interestingly, a study that compared platelet aggregation between patients with ovarian cancer and patients with benign tumors found no difference in platelet aggregation patterns [14]. There was no evidence of a reduced threshold of aggregation or hyperactivity between patients with ovarian cancer or patients with benign tumors.

Another anti-platelet agent with potential applicability in ovarian cancer prevention is DGLA, an essential omega-6 polyunsaturated fatty acid, and its metabolite, 12(S)-hydroxyeicosatetrienoic acid (12-HETrE), because they inhibit platelet aggregation with limited risk of bleeding [15]. Dietary DGLA and plasma levels of omega 6 fatty acids were found to be associated with reduced risk of breast and bladder cancers [16, 17]. In preclinical studies, DGLA reduced pancreatic and colon xenograft tumor weights and number of metastases in a delta 5 desaturase inhibition-dependent manners [18–20]. The mechanism of DGLA inhibition of cancer progression appears to involve indirect inhibition of metastasis tumor antigen [21].

DGLA is an essential fatty acid that is required at levels of 1–2% of total dietary fats for energy [22]. DGLA is obtained from its precursor gamma linolenic acid (GLA), which is found in high abundance in evening primrose, black currant and borage plant seed oils and fungal oil. GLA is metabolized in a rate-limiting reaction to DGLA by Δ-6- desaturase (D6D) [22]. Depending on the cell type, DGLA is further metabolized by cyclooxygenases or lipoxygenases to produce anti-inflammatory series 1 oxylipins (PG: PGD_1_, PGE_1_ and TXA_1_) and hydroxy-eicosatrienoic acids (12-HETrE and 15-HETrE), respectively [23]. DGLA can also be metabolized by delta-5-desaturase (D5D) to produce the inflammatory metabolite arachidonic acid (AA); however, humans and rodents have limited activity of this enzyme, hence smaller ratios of AA are produced from DGLA compared to PGs and HETrEs [22]. Inhibition of the D5D metabolism pathway has been shown to increase DGLA accumulation from 2.3 to 12% [22] and allow DGLA to inhibit pancreatic xenograft tumor growth [18]. High amounts of GLA (2.8 g/day for 42 days) were shown to be tolerated, with no side effects [24].

Taken together, these data suggest that antiplatelet agents such as DGLA may reduce the risk of ovarian cancer. In the current study, we developed a cell co-culture model to evaluate the interaction between ovarian cancer and platelets and tested the effect of DGLA on these interactions. We hypothesized that DGLA inhibits cancer cell viability in a platelet-dependent manner.

## Materials and methods

### Chemicals and drug compounds

DGLA (Cayman Chemical Company, 90230) was aliquoted, purged with argon gas and stored at ≤ -20°C. Freshly dissolved DGLA in molecular-grade ethanol or dimethyl sulfoxide (DMSO) was used in all experiments.

Cell culture-grade bovine serum albumin (BSA, 1992.9 mg) was resuspended in phosphate buffered saline (PBS, 20 ml) by vortexing, filtered using a 0.2 μm filter, and stored in 1.5 ml aliquots at -20°C. Fresh BSA/PBS control solution was used as control for DGLA treatment in cell culture.

### Collection and processing of platelets

De-identified platelet specimens were purchased from the Oklahoma Blood Institute (OBI). Also, platelets were collected from volunteers who provided written consent under the approved IRB #1426. No minors were included in this study. The OBI blood purchases and recruitment period for this study began on March 15, 2019 and ended on July 8, 2020. Butterfly 21G needles (BD 368656) were used to collect venous blood from all eligible participants who had signed an Informed Consent Form. Blood was directly collected in acid citrate dextrose (BD 364606) anticoagulant for a total of two tubes. The VetScan HM5 Hematology Analyzer by Zoetis in the primate species function or hemocytometer was used to count platelets. Centrifugation (1000 rpm, 10 min, room temperature without brakes) was used to separate platelet-rich plasma (PRP), the upper fraction, from red blood cells and other blood cells, which are the middle and lower fractions, respectively. Platelet poor plasma (PPP) control was collected by blood centrifugation (2000 rpm, 10 min, room temperature) and the upper fraction was collected.

### Cell culture

Ovarian cancer cell lines were purchased from ATCC, unless otherwise specified. OVCAR3 and OVSAHO cells were cultured in RPMI media supplemented with 10% fetal bovine albumin (FBS) and 1% penicillin/streptomycin solution. Human ES-2/GFP-luc (hereafter referred to as ES-2) and MES-OV/GFP-luc (hereafter referred to as MES-OV) were generated as described by Moisan et al. [25] and gifted by Dr. Branimir I Sikic at Stanford University. MES-OV and ES-2 cells were cultured in Dulbecco’s minimum essential medium (DMEM)/high glucose supplemented with 10%FBS and 1% penicillin/streptomycin solution. Fallopian tube secretory epithelial cultures (FT) were derived from healthy controls, as previously described [26], and cultured in DMEM-F12 media supplemented with 20% FBS and 1% penicillin/streptomycin solution.

### Effects of DGLA on cell viability

Amounts of epithelial cells (ovarian cancer cell lines, FT primary cultures, or an immortalized FT cell line) that would result in ~60% confluence within 24 h were plated on a 96-wells plate overnight to allow attachment. The next day, the cells were treated with different concentrations of DGLA (0 to 500 μM) or vehicle control (DMSO or ethanol) for 24 hours. After incubation, an (3-(4,5-dimethylthiazol-2-yl)-2,5-diphenyltetrazolium bromide/MTT assay (CellTiter 96 Aqueous Non-Radioactive Cell Proliferation Assay, Promega, Madison, WI, USA) was used according to the manufacturer’s instructions. Optical density (OD) was measured at wavelengths of 570 and 620 nm using a Synergy H1 microplate reader. The results were plotted, and doses that caused 50% of the maximal growth inhibition after normalization to the vehicle control were determined using GraphPad Prism 10.0.3.

### Spheroid formation

Multiple spheroids per well: Spheroids were generated as previously described. Briefly, approximately 80% confluent epithelial cells were trypsinized, harvested, washed, and resuspended in PBS. An optimized number of cells (10,000 to 50,000) was then cultured in a 96 well plate in 200 μL/well of spheroid media containing the base medium supplemented with commercially available 1x B27 (Gibco, 17504001), 20 ng/ml epidermal growth factor (Sigma-Aldrich E5036), 10 ng/ml basic fibroblast growth factor (Sigma-Aldrich, F0291), 5 μg/ml insulin(Sigma-Aldrich, I9278), and 0.4% BSA (Sigma-Aldrich, A9576). The plate was tightly covered with paraffin, and the spheres were imaged using a light microscope at 10x magnification.

3D Magnetic spheroid formation: A commercially available spheroid formation assay kit (Greiner BioOne) was used according to the manufacturer’s instructions. Briefly, 60% confluent FT cultures and ES-2 or MES-OV ovarian cancer cells were magnetically sensitized by co-incubating with NanoShuttle Biocompatible Nanoparticles 24 h prior to forming the spheres. Once cells achieved confluence in the wells, unbound nanoparticles were washed off and cells were cultured on 96 well plate on top of magnetic spheroid drive holders for 1–4 h to facilitate the formation of cell aggregates. Then, the magnets were removed and the resulting spheroids were evaluated. Oxford Optronix GelCount was used to image the spheroids and quantify their size and density.

### Platelet/epithelial cells co-cultures

Ovarian cancer cells (ES-2 and MES-OV) or healthy FT cultures were cultured in Transwell inserts for one week before imaging with a light microscope. Cancer cells were cultured inside the top insert and PRP was incubated in the bottom well.

### Flow cytometry

Annexin V binds to phosphatildyl serine molecules that translocate to the outer membrane during activation, hence Annexin V binding was used as a measure of platelets. A modified Annexin V-FITC Early Apoptosis Detection Kit (Cell signaling technology, 6592) was used to accomplish this purpose and was performed according to the manufacturer’s instructions. Briefly, platelets that had been co-incubated with epithelial cells were pelleted by centrifugation, washed with cold PBS, and resuspended in Annexin V Binding Buffer. Cells were co-incubated with Annexin V-FITC, Propidium Iodide on ice before analysis by flow cytometry.

### Western blot

Cells were plated on a 10 mm diameter plate for 24 h the day before treatment. When cells were approximately 60% confluent, they were treated with different doses of DGLA (0 to 250 μM) and incubated for 24 h. Mammalian protein extraction reagent (MPER, Thermo Fisher, 78501) was used to extract proteins from cell lysates or spheroids, according to the manufacturer’s instructions. Protein concentrations for each sample were estimated using Pierce BCA Protein Assay (Fisher Scientific, PI23225) according to the manufacturer’s instructions and 50 μg of protein was used for immunoblotting experiments Regulation of specific pathways was evaluated using primary and secondary antibodies. The following antibodies were purchased form Cells Signaling Technology, diluted according to the manufacturer’s instructions and used in immunoblotting: poly (ADP-ribose) polymerases (PARP 9542L or 5625S), death receptor 5 (DR5 8074S), cleaved caspase 3 (C-cas3 9662S), E-cadherin (14472S), N-cadherin (14215S), cyclophilin A (2175S), anti-rabbit secondary (7074S) and anti-mouse secondary (7076S).

### Statistical analysis

GraphPad Prism Software version 10.0.3 was used to perform all data analysis and statistical tests. Nonlinear regression was used to determine the DGLA half-maximal inhibitory concentration (IC_50_) in healthy cultures or ovarian cancer cells, linear regression was used to determine effect of increasing DGLA does on apoptosis and EMT markers, and ANOVA was used to compare multiple groups at the same time.

## Results

### DGLA inhibited healthy and cancer epithelial cell viability, and induced apoptosis only at doses ≥150 μM

To evaluate the direct effects of DGLA on epithelial cancer cell viability in culture, the MTT metabolic viability dye assay was conducted in triplicates to determine the IC_50_ of DGLA for four ovarian cancer cell lines (OVCAR3, MES-OV, OVSAHO and ES-2), an immortalized healthy fallopian culture (FT033 hTERT) and healthy FT primary culture (FT007, FT093 and FT100). FT cultures were used as normal cell control cultures, due to the evidence that the most common type of epithelial ovarian cancer, high grade serous, can arise from precursor lesions generated from secretory epithelial cells in fallopian tubes [27]. The IC_50_ values were within micromolar ranges between 136 μM and 279 μM (Table 1) regardless of cancer status.

**Table 1:** Average DGLA IC_50_ values of different epithelial cells. MTT assay was used to determine various ovarian cancer cells’ metabolic viability post-treatment with various concentrations of DGLA.

Cell Line	IC_50_ (μM)
OVCAR3	279(±20)
MES-OV	216(±70)
OVSAHO	136(±12)
ES-2	168(±27)
FT033 hTERT	235(±63)
FT	255(±80)

**Fig 1:**
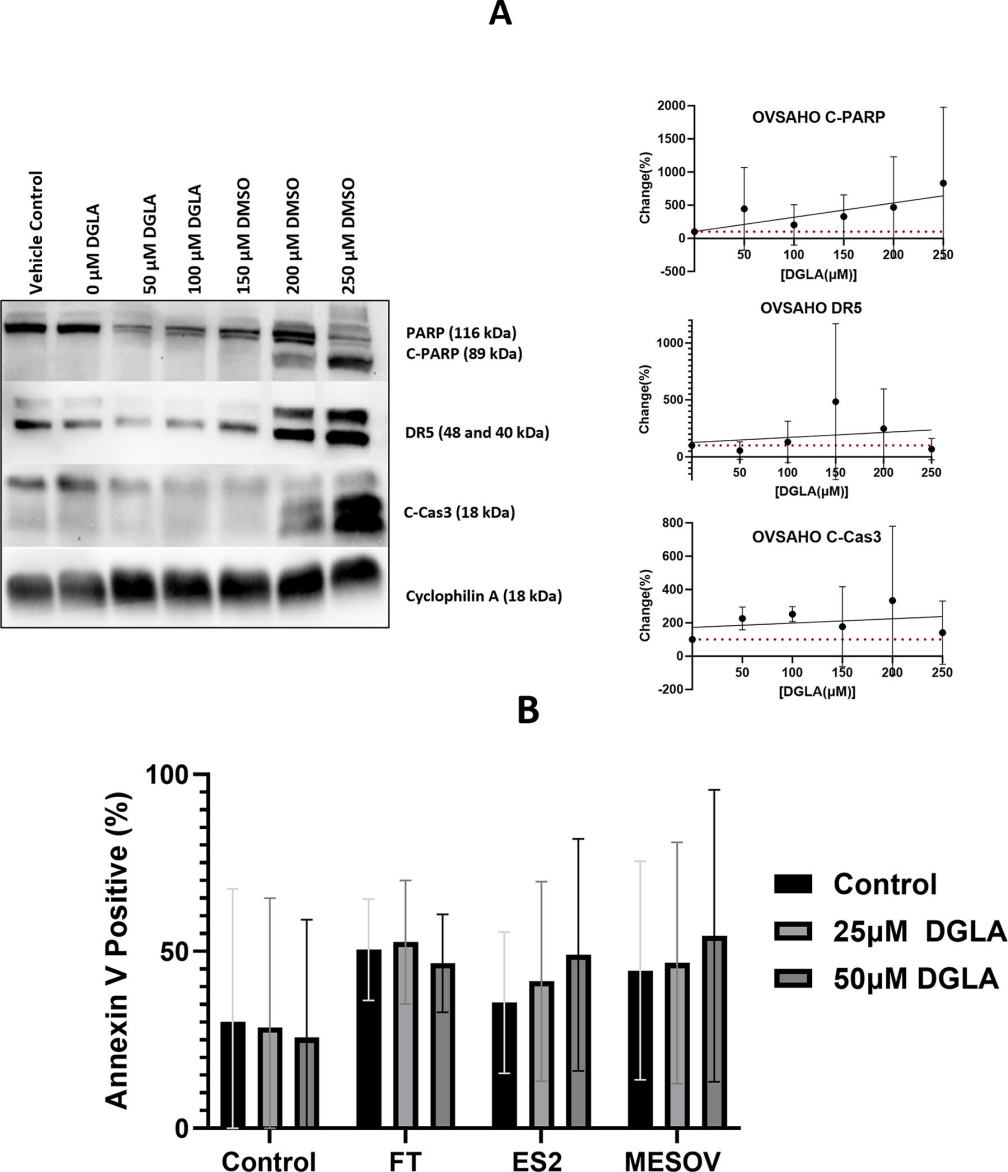
DGLA Effect of apoptosis markers. **A. Western blot analysis of DGLA effect on C-PARP, DR5, and C-Cas3 in ovarian cancer cells:** Ovarian cancer cells were treated with either vehicle control or different doses of DGLA (50–250 μM) for 24hrs. Western blots (representative image on left) analysis using total PARP, cleaved PARP, DR5, cleaved caspase 3 and cyclophilin A primary antibodies were quantified as shown on the right. **B. Flow cytometry analysis of phosphatidyl serine binding to Annexin V in platelets exposed to epithelial cells in presence of absence of DGLA:** Platelets were co-incubated with media control, FT or ovarian cancer cells that have been pre-treated with either vehicle control or DGLA (25 μM or 50 μM) for 90 minutes.

To study the cytotoxicity of DGLA at higher concentrations, markers of apoptosis were measured in protein extracts from four cancer cell lines (ES-2, OVCAR3, OVSAHO and MES-OV) treated with DGLA or vehicle control by western blot.). DGLA induced cleavage of poly-ADP ribose polymerase (PARP) and caspase 3 (Cas3), and increased expression of death receptor 5 (DR5) at concentrations equal to or above 150 μM (Fig 1A and S1 Fig). DR5 did not develop in ES2 western blot membranes. To confirm that DGLA does not cause apoptosis at lower doses, flow cytometry measurement of Annexin V staining of platelets was used to determine phosphatidyl serine translocation to the outer plasma membrane, as a marker of platelet activation. Platelets in the absence of cancer cells were used as a baseline control for outer membrane phosphatidyl serine expression. Co-incubation of platelets with the FT or ovarian cancer cells in inserts caused increased platelet activation and DGLA had minimal effects on this activation (Fig 1B).

### DGLA increased epithelial, and decreased mesenchymal, differentiation protein markers in cancer cells

**Fig 2:**
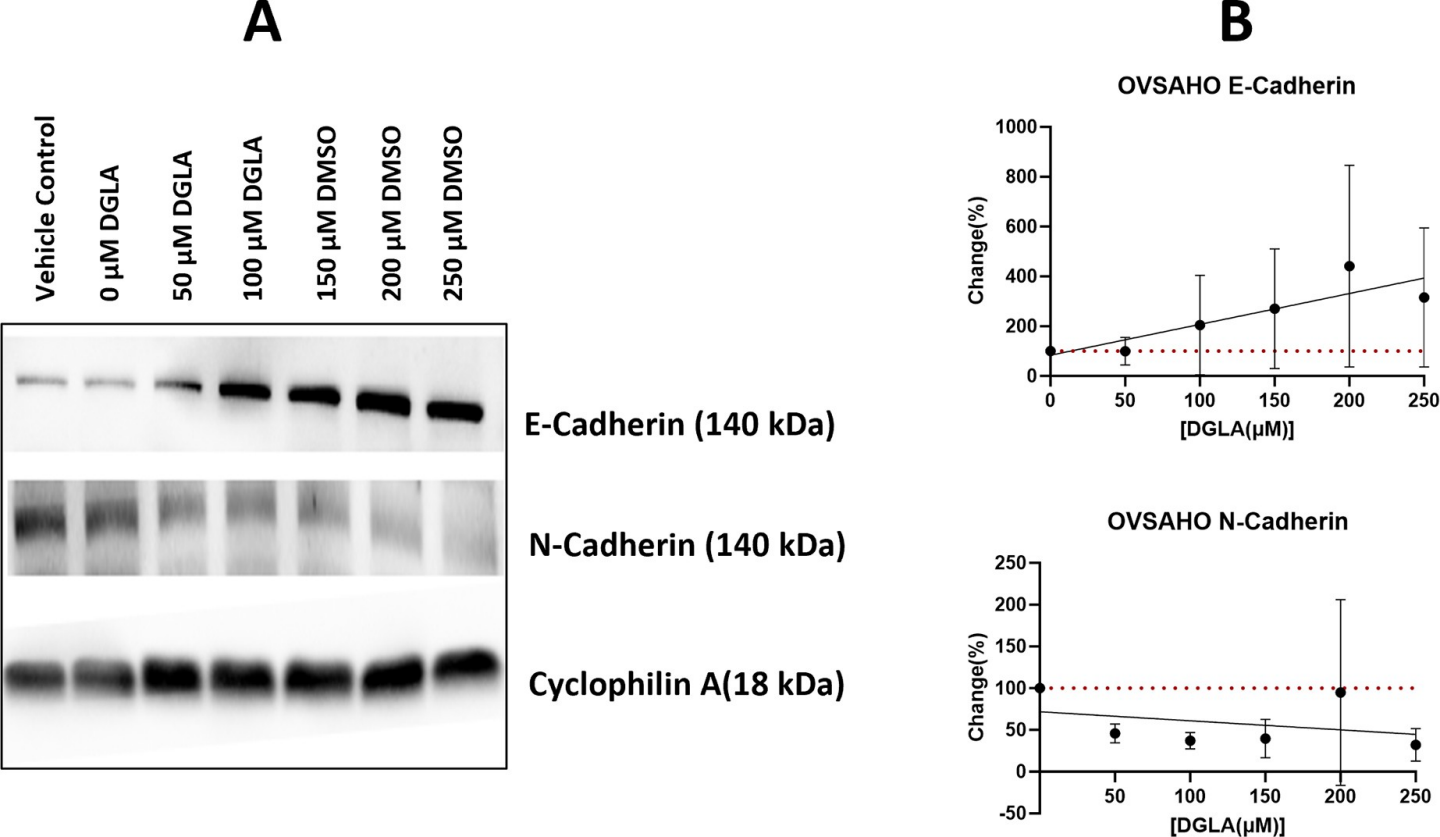
Western blot analysis of DGLA effect on EMT markers E-cadherin and N-cadherin in ovarian cancer cells. Ovarian cancer cells were treated with either DMSO control or different doses of DGLA for 24hrs. **A) Representative** western blot images of E-cadherin, N-cadherin and cyclophilin A. **B)** Quantification of Western blots images.

EMT is an important mechanism in cancer that promotes cell migration and metastasis to distant organs. To determine the effect of DGLA on this process, DGLA effects on expression of E-cadherin as an epithelial status marker and N-cadherin as mesenchymal status marker were measured by western blot using four cancer cell lines (ES-2, OVCAR3, OVSAHO and MES-OV). The results demonstrated a non-statistically significant DGLA dose-responsive increase in E-cadherin and a decrease in N-cadherin (Fig 2 and S2 Fig), suggesting that DGLA reverses EMT, hence making DGLA a valuable candidate to reduce metastases. This EMT reversal was observed across all DGLA doses with the greatest effects at 150 μM and above. Based on these results from apoptosis and EMT evaluation, all subsequent studies evaluating DGLA effects on the pathological interaction of platelets and ovarian cancer cells were done at concentrations ≤150 μM to avoid direct effects of DGLA on epithelial cells

### Platelets indirectly increase healthy and cancer epithelial cell spheroid size and density, and this interaction was not prevented by ≥150 μM DGLA

**Fig 3:**
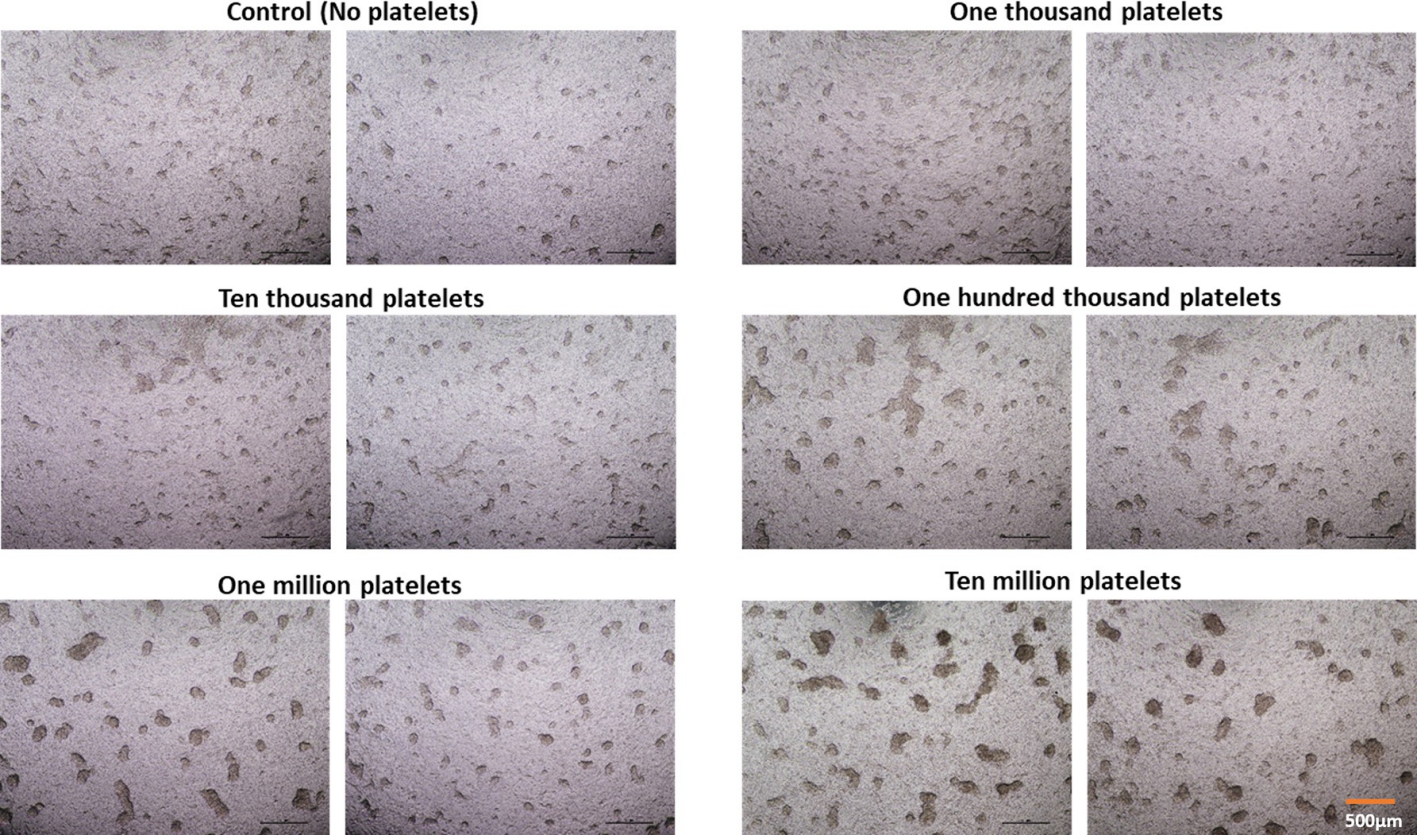
Effect of platelets on ES-2 ovarian cancer spheroids growth. Ovarian cancer cells in transwell inserts were co-incubated with media control or increasing numbers of platelets (1,000 to 10,000,000) in the wells for about a week. Spheroids were imaged with a light microscope to determine changes in spheroids’ size and shape.

Ovarian cancer is mostly diagnosed at a late stage (III/IV), when the disease has spread to the peritoneal cavity [28]. At these later stages, there is accumulation of peritoneal fluid called ascites, which contain ovarian cancer cell aggregates known as spheroids. To study the effects of platelets and DGLA on ovarian cancer spheroids, we modified a model that generates multiple spheroids per well during a week of incubation, as previously described [29]. Then Transwell inserts were utilized to co-culture platelets with spheroids generated from healthy FT, a clear cell carcinoma ES-2 cell line and a high grade serous ovarian carcinoma cell line MES-OV in separate physical compartments, which allowed transmission of small molecules, but no physical contact, between epithelial cells and platelets. Platelets were cultured in the bottom well, and spheroids were placed in a Transwell insert containing a 0.4 μm pore size filter. After one week of incubation, platelet number dependent-induced increases in spheroid sizes were observed (Fig 3).

**Fig 4:**
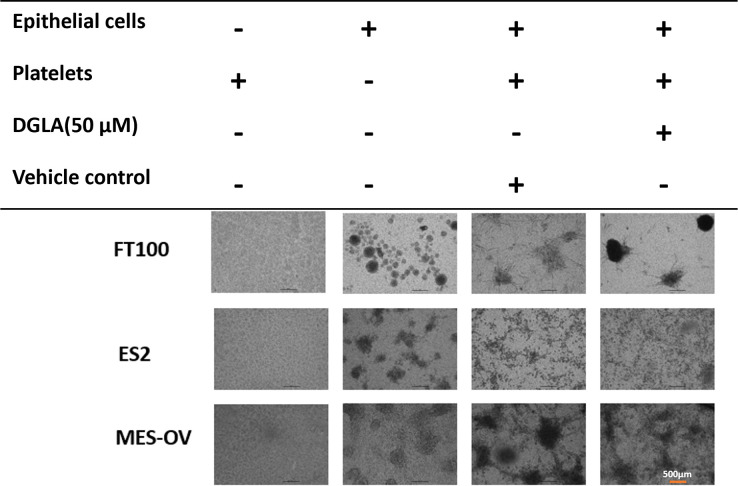
Effect of DGLA on epithelial cells spheroids formation. FT, ES-2 or MES-OV ovarian cancer cells were co-incubated with media control or 10,000,000 n platelets in transwell insert for about a week. A light microscope was used to visualize formation of spheroids and changes in spheroids’ size and shape.

To test if DGLA altered platelet effects on FT or cancer spheroids, DGLA (50 μM) was added to the bottom wells of platelet-ovarian cancer cell co-culture setups right before adding platelets. DGLA (50 μM) addition to the bottom wells of these co-culture experiments had no effects on spheroid sizes regardless of cancer status (Fig 4 and S3 Fig).

**Fig 5:**
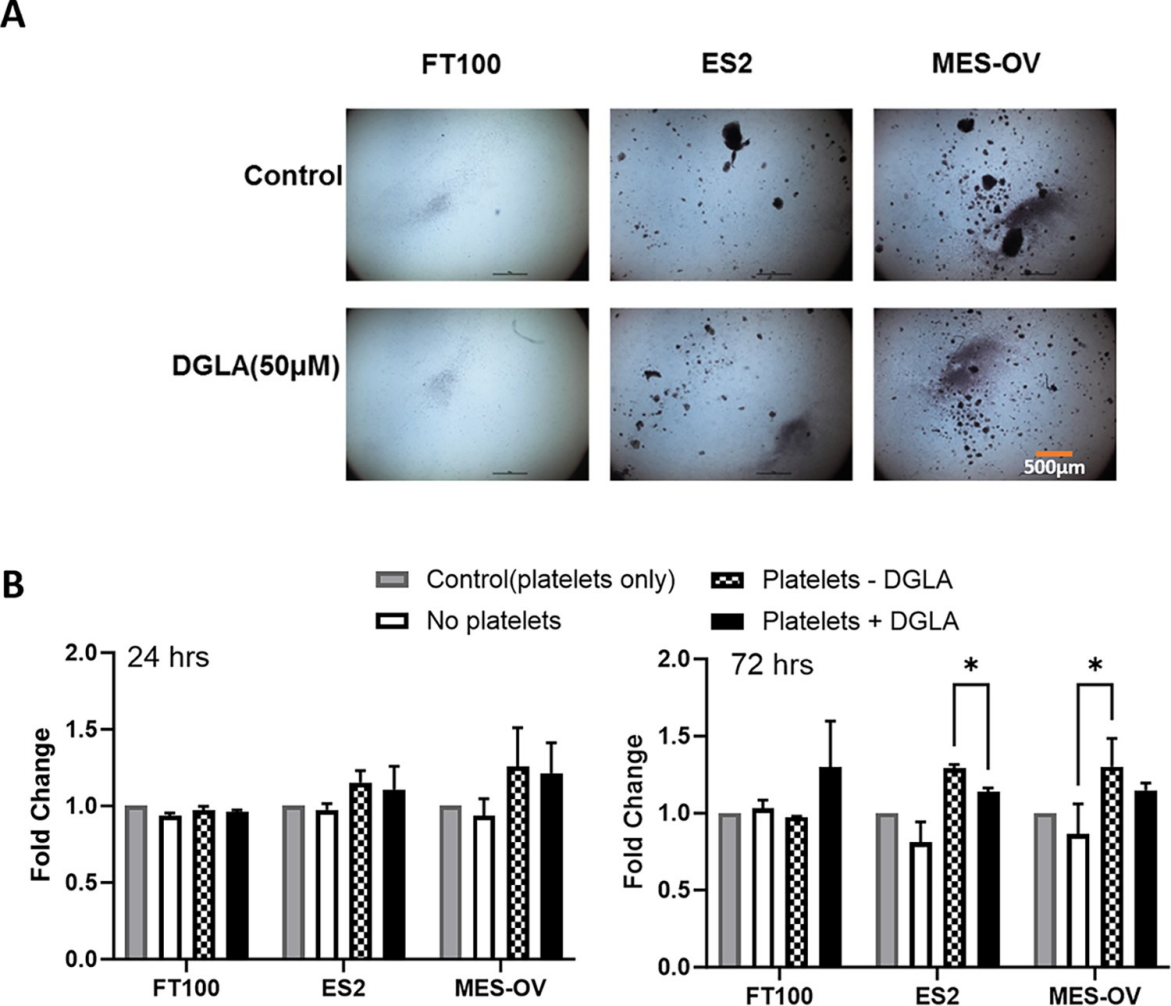
Effect of DGLA on epithelial cells-mediated platelets clumping. **A.** Representative images of platelets aggregates. **B.** Quantification of platelets aggregates. Ovarian cancer spheroids in transwell inserts were co-incubated with media control or various number of platelets in the wells. Platelets were imaged with a light microscope to determine platelet clumps formation and clump size after 24 or 72 hrs.

Examination of the platelets in the bottom wells demonstrated that the two different ovarian cancer cell lines (ES-2 and MES-OV), but not healthy FT cells, induced visible platelet aggregates. DGLA slightly decreased platelets clump formation after 72 hours of treatment, but the effect was not statistically significant (Fig 5). There was no effect of the DGLA treatment.

### Platelets directly caused compression of healthy and cancer epithelial cell spheroids, and this interaction was not prevented by 25 μM DGLA

**Fig 6:**
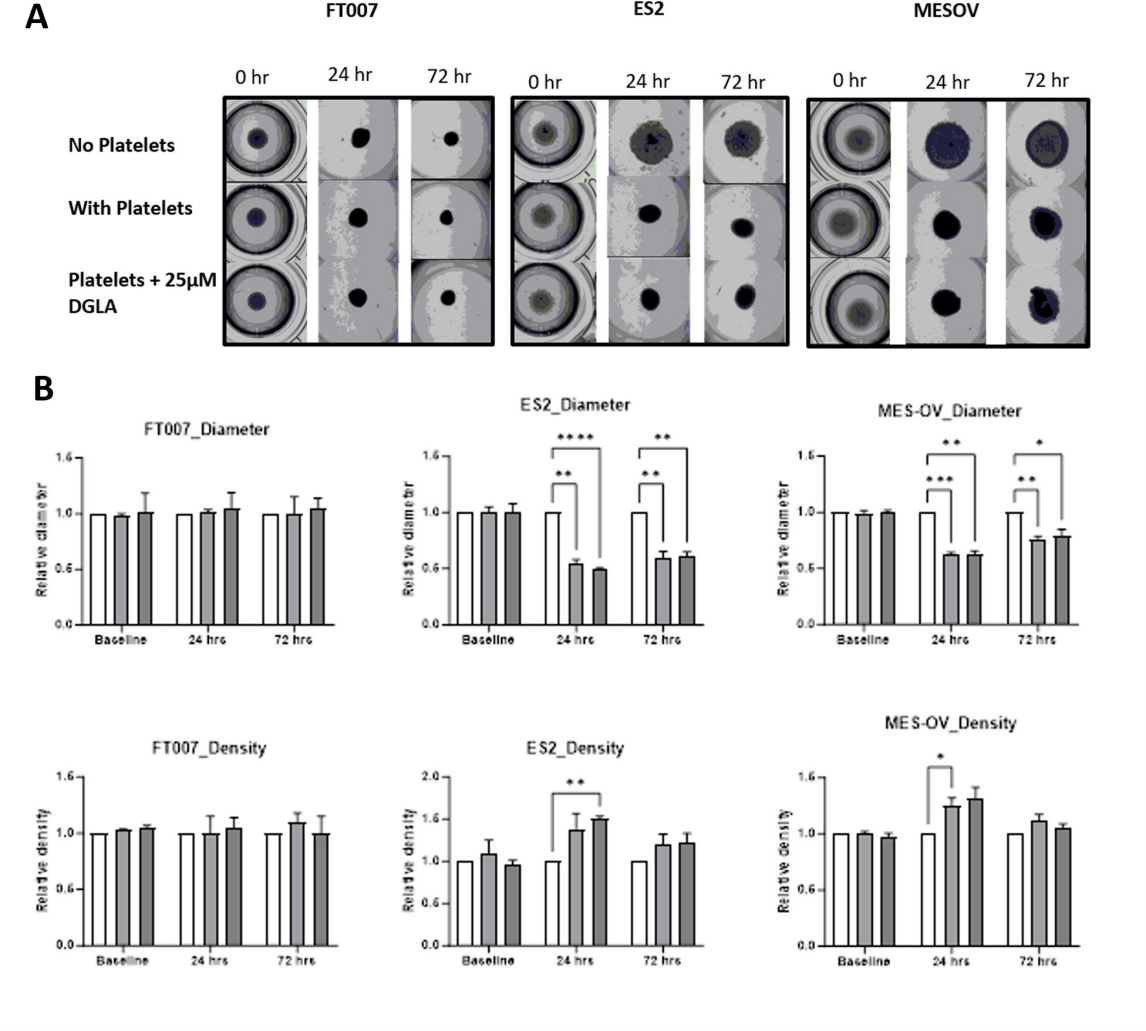
Effect of platelets and DGLA on magnetic spheroids size and density. **A)** FT or ovarian cancer (ES-2 and MES-OV) spheroids generated with magnetic nanoparticles and simultaneously co-incubated with/without platelets and DMSO vehicle control or DGLA. **B)** Quantification of spheroid diameter and density in images created with Oxford Optronix GelCount.

To model direct interactions between platelets and epithelial cells, platelets were added directly to spheroids generated with a commercially available magnetic bioprinting protocol that forms one spheroid per well. Cells harboring magnetic nanoshuttles were exposed to a magnet so that they instantly formed spheres and then were incubated with or without platelets. Co-incubation with platelets caused compression of the spheroids (decreased size and increased density) for spheroids formed by cancer cells, but not for healthy FT cultures. To test if DGLA altered these platelet-mediated changes, low dose DGLA (25 μM) was added to the spheroid right before addition of platelets. DGLA (25 μM) had no effect on the platelet induced spheroid compression at 24 or 72 hours of treatment (Fig 6).

## Discussion

Cancer and platelets have been shown to promote each other in a pathologic feedforward loop that aggravates the poor prognosis of ovarian cancer due to high mortality caused by both ovarian cancer and thrombosis. Thrombocytosis is reported as a significant cause of mortality in ovarian cancer [30] and in other cancers, including gastric [31, 32], colon [33, 34], lung [33, 35] and esophageal [36, 37]. Up to 30% of ovarian cancer patients have thrombocytosis at diagnosis, which is associated with both reduced progression free and overall survival [30]. Thrombotic events occur in up to 6.8% of ovarian cancer patients and this risk is highest in patients with clear cell carcinoma histology, which is associated with a 14.5–27.3% thrombotic events rate [38, 39]. In fact, patients with ovarian clear cell carcinoma often are diagnosed with deep vein thrombosis or pulmonary emboli at the time of their diagnosis or before they undergo cytoreductive surgery [38–41], suggesting the thrombotic events are not due to cancer treatment. However, there have been reports of thrombotic events among ovarian clear cell carcinoma patients with recurrent disease [39], suggesting that surgery and chemotherapy further increase the risk of thrombotic events in these patient populations. In epithelial ovarian cancer, reduction of platelet count by no more than 25% post-treatment was shown to be significantly associated with reduced progression free survival and reduced overall survival [42], supporting that platelets might serve as targets for secondary ovarian cancer prevention strategies, such as using platelet inhibitors in maintenance therapy to prevent recurrence after primary treatment is complete.

In this study, we demonstrated that the pathological interactions of platelets and ovarian cancer cells can be modeled in cell culture, and that DGLA has no effects on these interactions. Both healthy and cancer cells caused platelet aggregation, however platelets only affected spheroid formation by cancer cells and had no effect on healthy epithelial cell spheroid formation. This effect of platelets on ovarian cancer cell spheroid formation was observed in two independent models. In the first model, the spheroids were co-incubated with platelets using a transwell insert that prevented direct contact of the platelets with the spheroids. In the second model, the platelets were directly incubated with spheroids formed using magnetic nanoshuttles and a magnet to gather the cancer cells in close proximity.

We have shown that DGLA, in the absence of platelets, reduced epithelial cell viability at IC_50_’s in the 150–275 μM range. Within this concentration range, DGLA appeared to induce apoptosis in ovarian cancer cells as shown by an increase in PARP and caspase 3 cleavage and upregulated DR5 expression, which helps to explain mechanism of reporter DGLA anticancer activity in multiple cancers. To better understand the effect of platelets and DGLA effect on platelets, we developed spheroid models that evaluate directly physical interactions and indirect physical interactions between platelets and cancer cells. Using these models, we have shown that physiological concentrations of DGLA do not alter either the effects of platelets on cancer spheroids or the effect of cancer spheroids on platelets. Furthermore, DGLA toxicity was not limited to cancer cells or platelets; it affected both cancer cells and healthy cultures equally with no selectivity. The effect of the major metabolite of DGLA, 12-HETrE, on platelets occurs through the prostacyclin receptor [43], suggesting that this receptor is not involved in the mechanism of platelet and ovarian cancer cell interaction. Taken together, these data suggest that DGLA might be a good candidate to inhibit cancer growth, however its mechanism is not directly specific to platelets, hence making it a less attractive candidate for limiting interactions between cancer cells or platelets. Therefore, there is a high risk of toxic side effects on normal cells when DGLA is used at high concentrations. Furthermore, the concentrations at which DGLA caused toxic effects are higher than maximal-achievable plasma concentration, suggesting that DGLA’s action might be a result of its major metabolites, which are more specific towards cancer.

DGLA metabolites and byproducts (PGE1, 15-HETrE and 8-hydroxyoctanoic acid) also possess anticancer activity as they activate protein kinase C (PKC) signaling pathway or act as histone deacetylase (HDAC) inhibitors, hence leading to cell cycle arrest or apoptosis [18, 22]. Although prostate cancer studies suggest no improvement in malignant disease with higher levels of plasma or tissue DGLA, high arachidonic acid levels were shown to be highly associated with malignant disease [44]. These data suggest future studies that test the potential interference with platelet/ovarian cancer cell interactions by DGLA metabolites that are known to have antiplatelet effects, such as 12HETrE. The effect of these metabolites should first be validated for their selectivity either on cancer cells while sparing healthy cells, or selectivity towards platelets alone without harming normal epithelial cells to avoid toxicity.

In summary, the pathologic interaction of platelets and ovarian cancer cells can be observed in cell culture, however DGLA treatment does not interfere with this interaction. While these results support development of anti-platelet strategies for prevention and treatment of ovarian cancer and reduction of thrombosis risk in ovarian cancer patients, they suggest that DGLA is not a rational candidate in this endeavor.

## Supporting information

S1 Fig DGLA Effect of apoptosis markers. Ovarian cancer cells (ES-2, OVCAR-3, MES-OV and OVSAHO) were treated with either vehicle control or different doses of DGLA for 24hrs. Densitometry of western blots performed using cleaved PARP, DR5, cleaved caspase 3 and cyclophilin A (as a housekeeping gene) primary antibodies was done to quantify protein in each sample. DR5 was not detected in ES2 cells.
(TIF)

S2 Fig Western blot analysis of DGLA effect on EMT markers E-cadherin and N-cadherin in ovarian cancer cells. Ovarian cancer cells (ES-2, OVCAR-3, MES-OV and OVSAHO) were treated with either vehicle control or different doses of DGLA for 24hrs. Western blots performed using antibodies to E-cadherin, N-cadherin, or cyclophilin A (as a housekeeping gene) were quantified.
(TIF)

S3 Fig Effect of DGLA on epithelial cells spheroid formation. ES-2 and MES-OV ovarian cancer cells were co-incubated with media control or 10,000,000 platelets in transwell inserts for about a week. A light microscope was used to visualize formation of spheroids and changes in spheroids’ size and shape.
(TIF)

S1 Raw images (PDF)
