# Supplementary figures and images for: Platelet and epithelial cell interations can be modeled in cell culture, and are not affected by dihomo-gamma-linolenic acid
Source: PLoS One. 2024 Aug 27;19(8):e0309125. doi: 10.1371/journal.pone.0309125 (PMC11349180; doi:10.1371/journal.pone.0309125)

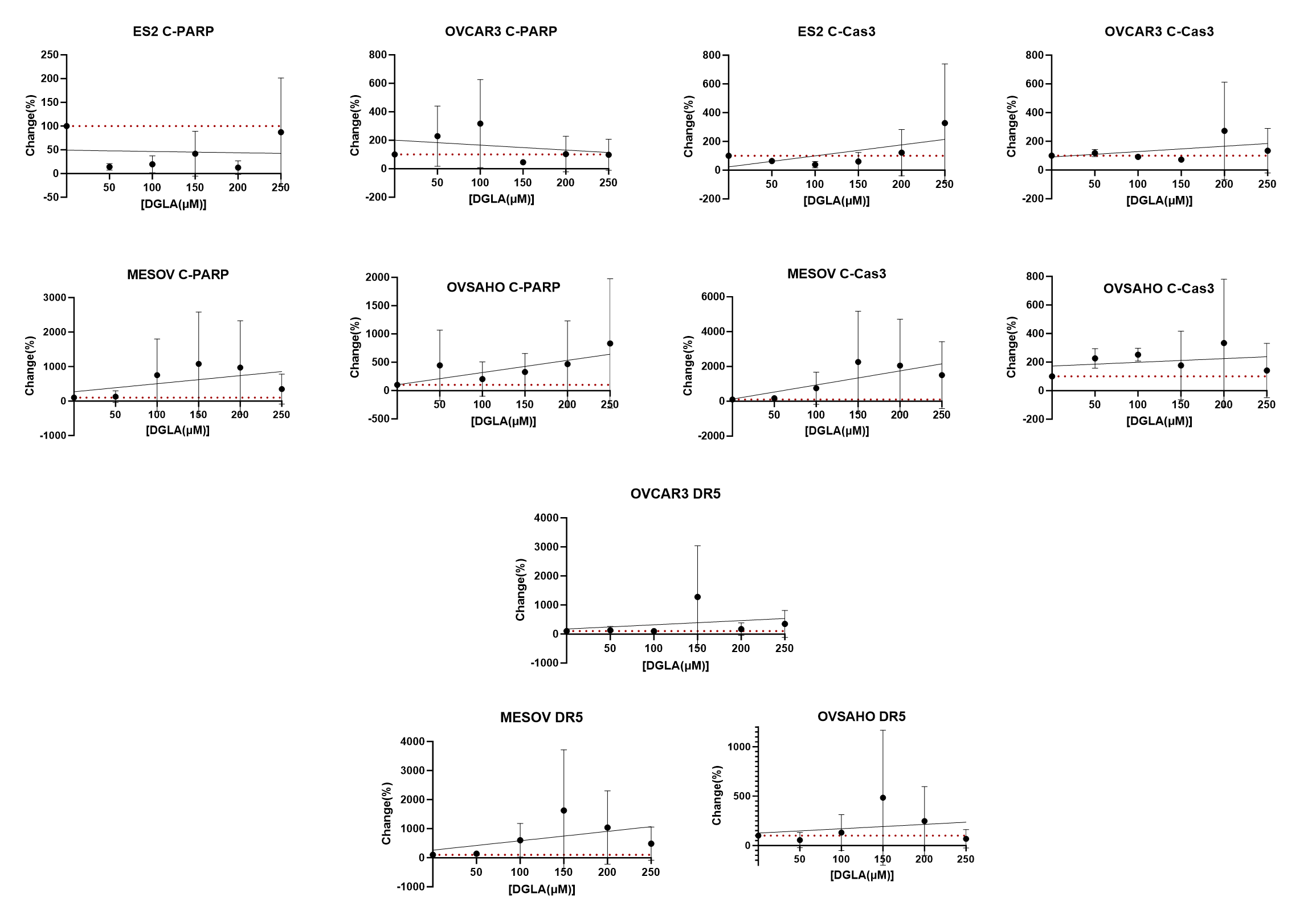

Supplement: S1 Fig — Ovarian cancer cells (ES-2, OVCAR-3, MES-OV and OVSAHO) were treated with either vehicle control or different doses of DGLA for 24hrs. Densitometry of western blots performed using cleaved PARP, DR5, cleaved caspase 3 and cyclophilin A (as a housekeeping gene) primary antibodies was done to quantify protein in each sample. DR5 was not detected in ES2 cells. (TIF) [file pone.0309125.s001.tif]

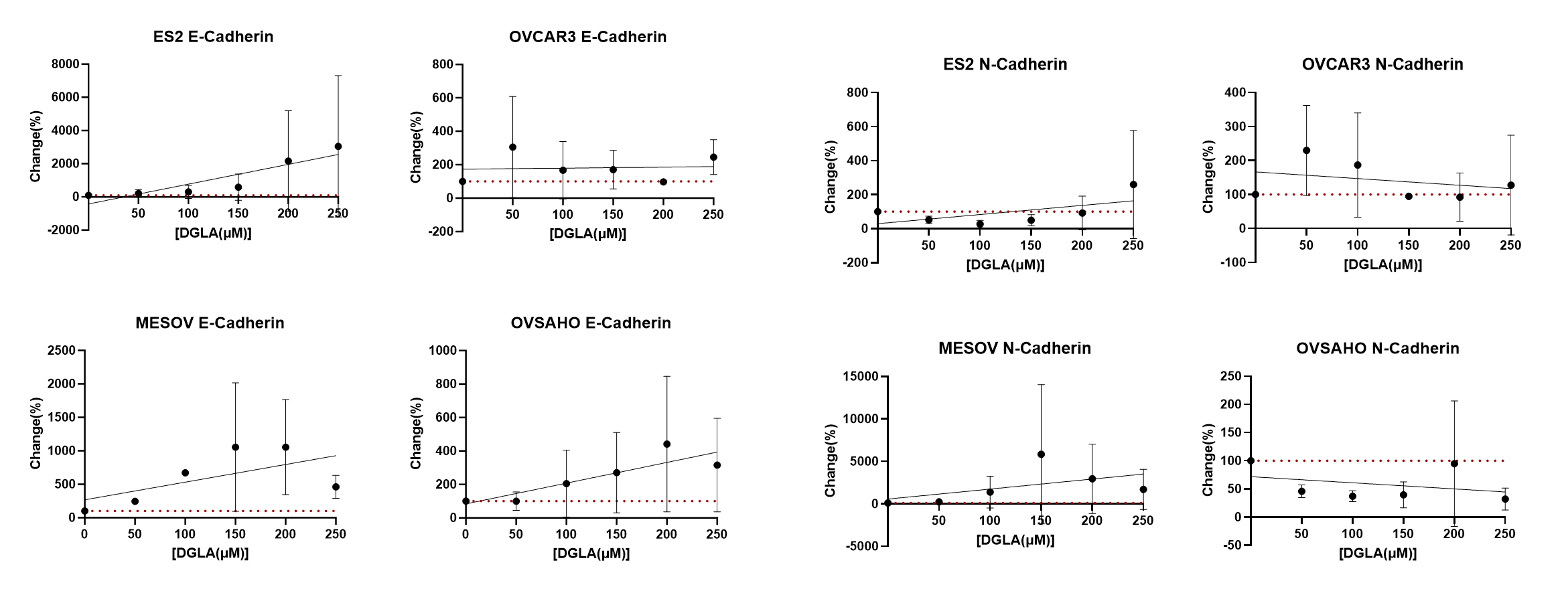

Supplement: S2 Fig — Ovarian cancer cells (ES-2, OVCAR-3, MES-OV and OVSAHO) were treated with either vehicle control or different doses of DGLA for 24hrs. Western blots performed using antibodies to E-cadherin, N-cadherin, or cyclophilin A (as a housekeeping gene) were quantified. (TIF) [file pone.0309125.s002.tif]

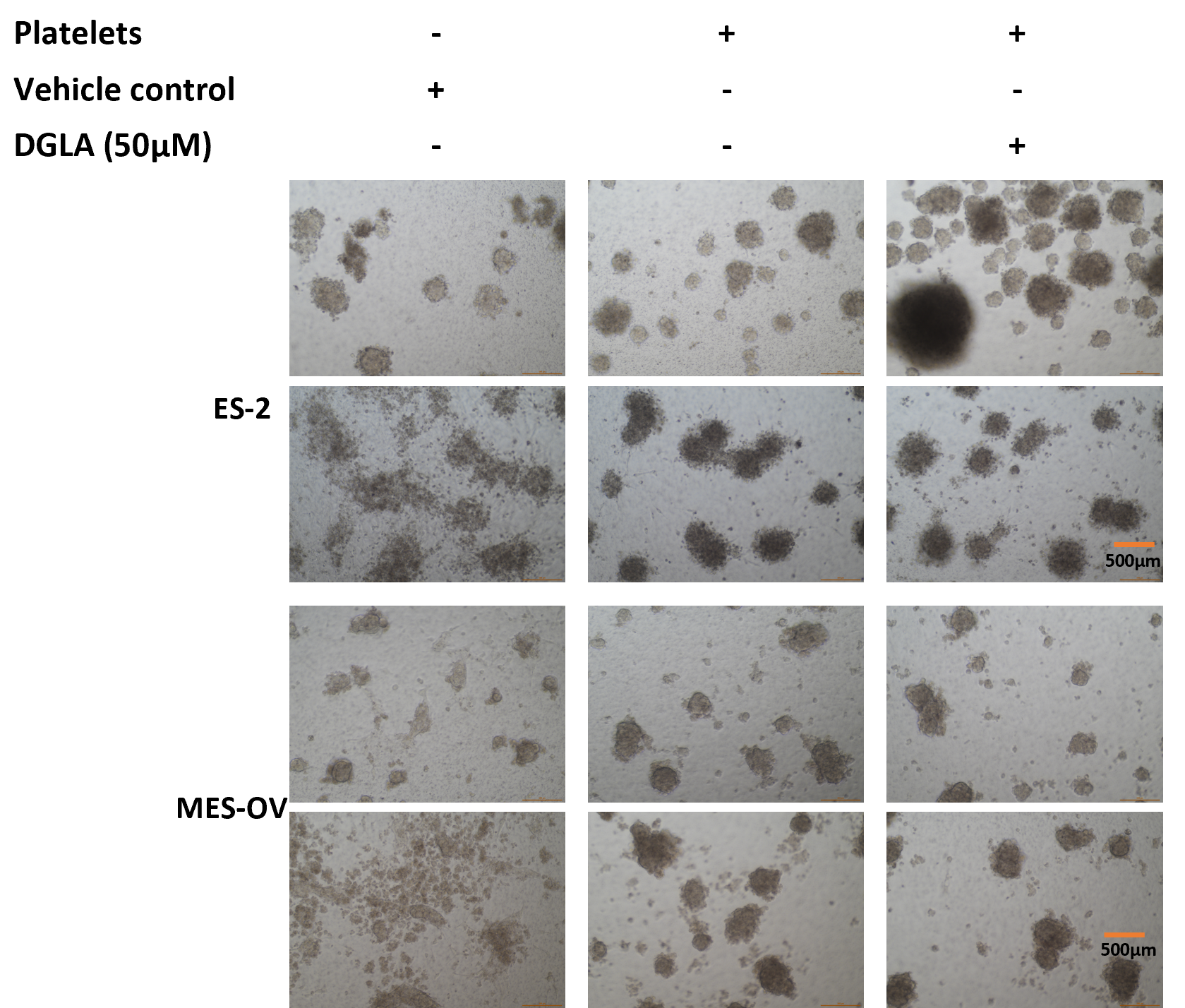

Supplement: S3 Fig — ES-2 and MES-OV ovarian cancer cells were co-incubated with media control or 10,000,000 platelets in transwell inserts for about a week. A light microscope was used to visualize formation of spheroids and changes in spheroids’ size and shape. (TIF) [file pone.0309125.s003.tif]

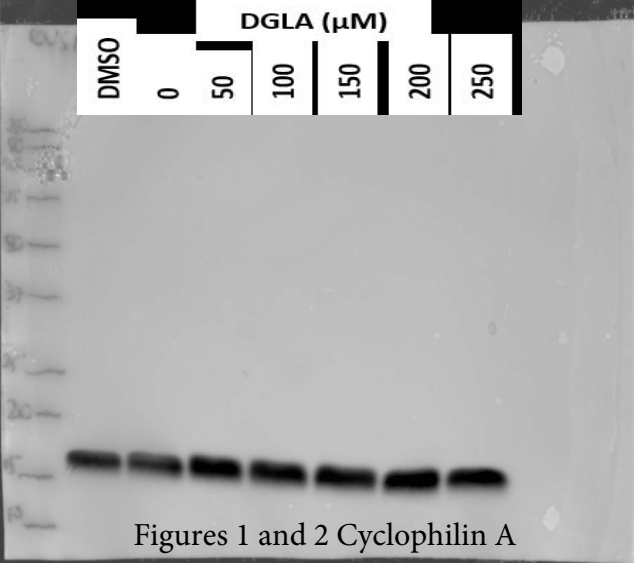

Figures 1 and 2 Cyclophilin A

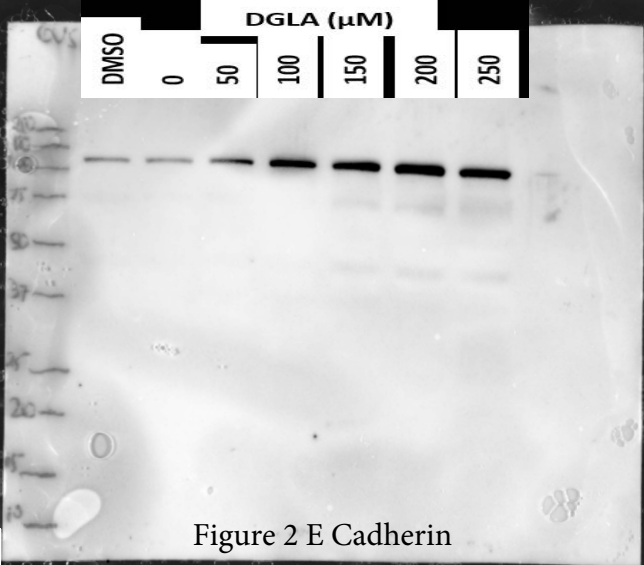

| DGLA ( $\mu$ M) |   |    |     |     |     |     |
|-----------------|---|----|-----|-----|-----|-----|
| DMSO            | 0 | 50 | 100 | 150 | 200 | 250 |

35  
 30  
 25  
 20  
 15  
 10

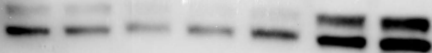

Figure 1 Death Receptor 5

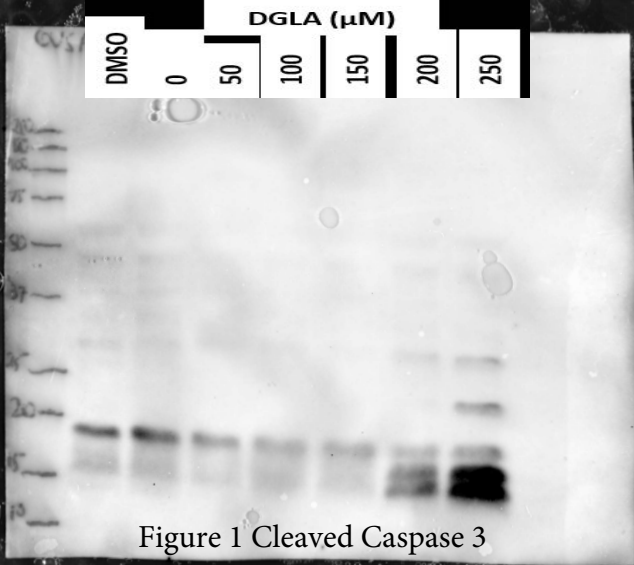

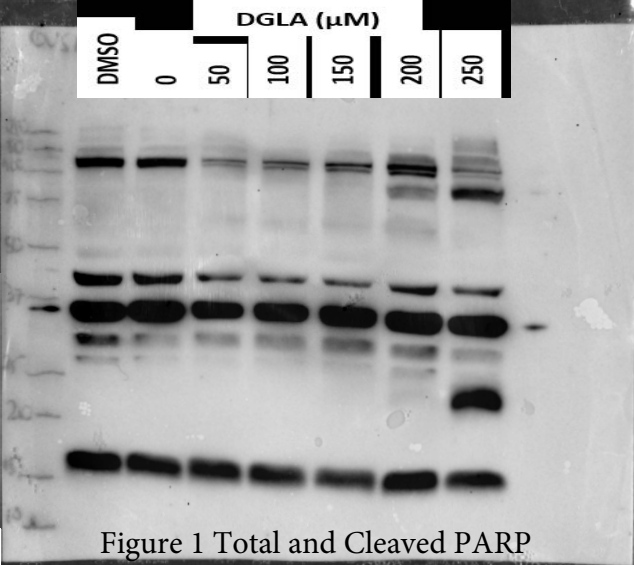

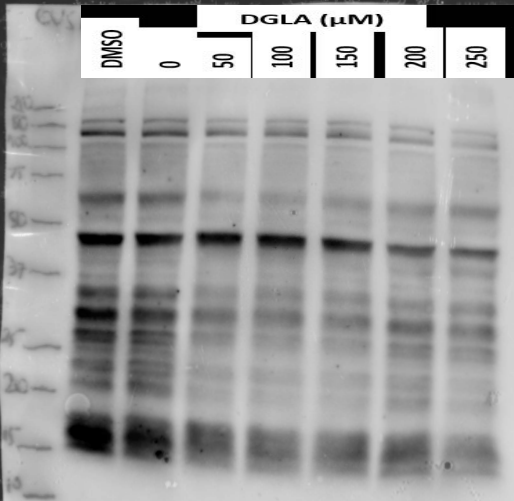

Figure 2 N Cadherin

Supplement: S1 Raw images — (PDF) [file pone.0309125.s004.pdf]
